# Photocatalytic Activity of Ag Nanoparticles Deposited on Thermoexfoliated g-C_3_N_4_

**DOI:** 10.3390/nano14070623

**Published:** 2024-04-02

**Authors:** Karina Portillo-Cortez, Uriel Caudillo-Flores, Perla Sánchez-López, Elena Smolentseva, David Dominguez, Sergio Fuentes-Moyado

**Affiliations:** Centro de Nanociencias y Nanotecnología, Universidad Nacional Autónoma de México, Ensenada CP 22860, Mexico; portillo@ens.cnyn.unam.mx (K.P.-C.); perlasanchez@ens.cnyn.unam.mx (P.S.-L.); elena@ens.cnyn.unam.mx (E.S.); david@ens.cnyn.unam.mx (D.D.); fuentes@ens.cnyn.unam.mx (S.F.-M.)

**Keywords:** photocatalysis, Ag/g-C_3_N_4_, nanocomposites, dye degradation, visible, exfoliated g-C_3_N_4_

## Abstract

The limited access to fresh water and the increased presence of emergent pollutants (EPs) in wastewater has increased the interest in developing strategies for wastewater remediation, including photocatalysis. Graphitic carbon nitride (g-C_3_N_4_) is a 2D non-metal material with outstanding properties, such as a 2.7 eV bandgap and physicochemical stability, making it a promising photocatalyst. This work reports the process of obtaining high-surface-area (SA) g-C_3_N_4_ using the thermal-exfoliation process and the posterior effect of Ag-nanoparticle loading over the exfoliated g-C_3_N_4_ surface. The photocatalytic activity of samples was evaluated through methylene blue (MB) degradation under visible-light radiation and correlated to its physical properties obtained by XRD, TEM, BET, and UV–Vis analyses. Moreover, 74% MB degradation was achieved by exfoliated g-C_3_N_4_ compared to its bulk counterpart (55%) in 180 min. Moreover, better photocatalytic performances (94% MB remotion) were registered at low Ag loading, with 5 wt.% as the optimal value. Such an improvement is attributed to the synergetic effect produced by a higher SA and the role of Ag nanoparticles in preventing charge-recombination processes. Based on the results, this work provides a simple and efficient methodology to obtain Ag/g-C_3_N_4_ photocatalysts with enhanced photocatalytic performance that is adequate for water remediation under sunlight conditions.

## 1. Introduction

Access to clean water is essential for human survival, but only a tiny fraction (0.3%) of the world’s freshwater is suitable for daily use [1]. With the increase in the global population, new challenges have arisen, such as higher demand for freshwater and the emergence of highly persistent and chemically stable contaminants, also known as emerging contaminants (ECs). The latter are becoming increasingly concerning, as these contaminants are difficult to eliminate using conventional treatment processes, and their presence in water bodies is not strictly regulated. As a result, we might face a shortage of freshwater resources, which will negatively impact human and environmental health due to exposure to these contaminants. Among ECs, dyes are a type of contaminant whose use can extend to various industries (textile, art, food, construction, etc.), but they are also a significant source of water pollution. Approximately 15,000 tons of the worldwide production of dyes is directly discharged into water, reducing the self-purification ability of water systems [1,2]. Therefore, the scientific community worldwide is working together to provide alternative and efficient strategies to treat water, ensuring that we have access to this vital natural resource.

Numerous strategies have been proposed to remove organic contaminants, including adsorption, filtration, coagulation, biosorption, ion exchange, and advanced oxidation processes (AOPs). Among these AOPs, photocatalysis is a low-cost and environmentally friendly technology that has gained attention in wastewater treatment due to its high efficiency [3,4]. Photocatalysis involves the activation of a semiconductor material by light energy, which removes organic pollutants from water through a series of non-selective redox reactions caused by highly oxidizing species such as superoxide (^•^O_2_^−^) and hydroxyl (^•^OH) radicals [5]. Metal oxide semiconductors, such as TiO_2_, ZnO, and ZnS, have been widely studied as photocatalysts, with excellent performance. However, they required UV radiation to function [6,7].

Efforts are being made to find materials that can facilitate redox reactions under visible light or sunlight radiation, as they make up a significant portion of the solar spectrum (45%) compared to UV light (4%) [5]. Graphitic carbon nitride (g-C_3_N_4_) is a promising metal-free material employed in the photo-splitting of water, the remotion of organic pollutants, and photosynthesis under visible radiation. Moreover, g-C_3_N_4_ is the most stable allotrope of carbon nitride, composed of tri-s-triazine subunits connected through planar tertiary amino groups in a layer. This material exhibits a bandgap of ca. 2.70 eV, is cost-effective, has good chemical stability, is easily accessible, and is environmentally friendly [5,8,9]. However, g-C_3_N_4_ in bulk form has a poor specific surface area and a fast electron-hole pair recombination rate, leading to low photocatalytic performance [10]. Several strategies have been employed to address these drawbacks and improve the properties of g-C_3_N_4_, including metal or non-metal doping [11,12,13], noble metal decoration [14,15], hetero-structuring by coupling with other semiconductors [16,17], surface modification, and surface area (SA) incrementing [18].

Several works in the literature have described various methodologies for incrementing the surface area of g-C_3_N_4_ [19]. Ultrasonic exfoliation results in surface areas of ~130 m^2^ g^−1^, while chemical exfoliation can yield values up to 200 m^2^ g^−1^. However, these processes are often complicated and require additional steps. On the other hand, thermal exfoliation is a simple methodology, but the results reported are usually low surface areas of less than 80 m^2^ g^−1^. Therefore, it is necessary to obtain higher surface-area values using this simple methodology to improve the photocatalytic activity, as has been proved by chemical and ultrasonic exfoliation methods [3,20,21,22].

Another approach to enhance the photocatalytic performance of g-C_3_N_4_ is metal deposition, as photogenerated charge carriers can be effectively separated [5,13,15,23]. When g-C_3_N_4_ comes into contact with metal, a metal–semiconductor heterojunction can be formed, capturing the photogenerated electron and preventing electron-hole recombination. The type of heterojunction formed (Schottky barrier or ohmic contact) depends on the relative work function levels of g-C_3_N_4_ and the metal [24,25]. Noble metals such as Pt and Pd perform well when they decorate semiconductors [1,26,27]; however, their use is expensive. Silver (Ag) is a metal with extensive applications in biosensors, biomedicine, and catalysis. The integration of Ag metal over photocatalysts has acquired great interest, owing to the effect of both surface plasmon resonance (SPR) and the fact that it can act as an electron trap. It has been reported that photocatalyst materials based on Ag and g-C_3_N_4_ (i.e., Ag/g-C_3_N_4_ nanocomposite) exhibit high performance in degrading different dyes like methyl orange (MO), methylene blue (MB), and rhodamine B (RhB) [28,29,30,31] under visible radiation.

Therefore, this study aims to evaluate the photocatalytic activity of a series of nanocomposites based on g-C_3_N_4_ thermal-exfoliated nanosheets with a high surface area (>100 m^2^ g^−1^) decorated with different percentages of Ag. Combining the results of the photocatalytic and scavenger activity tests with the results of the ex situ characterization, specifically the handling and recombination of charge carriers by the composite materials, it was possible to determine the causes that allowed us to improve the photoactivity of the nanocomposites (Ag/g-C_3_N_4_) compared to pure materials.

## 2. Materials and Methods

### 2.1. Materials Synthesis

Graphitic carbon nitride was prepared using the conventional thermal polymerization process of dicyandiamide (99%, Sigma-Aldrich, St. Louis, MO, USA). The overall process consisted of a two-step calcination procedure; the first calcination was carried out at 500 °C for 2 h. Then, the obtained material was again calcinated at 500 °C for 2 h using a heating ramp of 4 °C min^−1^ to obtain the final exfoliated g-C_3_N_4_. Silver nanoparticles were incorporated into g-C_3_N_4_ using a deposition method with varying amounts (1, 5, 10, and 15 wt.%) of an AgNO_3_ (Sigma-Aldrich) solution. The support material was suspended in a deionized water solution for 30 min, to which the appropriate amount of AgNO_3_ was added and stirred for an additional 5 min. The reduction was carried out using a NaBH_4_ (Sigma-Aldrich) aqueous solution with an Ag/NaBH_4_ molar ratio of 1/5. The final solid was profusely rinsed with deionized water, collected by centrifugation, and dried at 80 °C. The samples were labeled as xAg/g-C_3_N_4_, with x indicating the amount (wt.%) of Ag deposited over the g-C_3_N_4_.

### 2.2. Materials Characterization

The crystalline structure of g-C_3_N_4_ and Ag/g-C_3_N_4_ photocatalysts was determined by X-ray powder diffraction (XRD) in a Panalytical-Aeris diffractometer (Malvern Panalytical B.V., Almelo, The Netherlands). Monochromatic CuKα radiation (λ = 1.54184 Å) was used with a 2θ angle ranging from 5° to 80° and a step size of 0.01° with a time of 1 s per step. The morphology of the silver NPs on the g-C_3_N_4_ surface was analyzed by HRTEM using a JEOL JEM-2010 microscope (JEOL Ltd., Tokyo, Japan). Textural properties of the obtained samples were determined through N_2_ adsorption–desorption isotherms recorded at 196 °C using Micromeritics TriStar 3000 equipment (Micromeritics Instruments Corp., Norcross, GA, USA). The specific surface area of the samples was estimated using the Brunauer–Emmertt–Teller (BET) method and nitrogen adsorption data within the P/P0 range of 0.005–0.250. The average pore diameter was calculated by applying the Barrett–Joyner–Halenda (BJH) method to the adsorption and desorption branches of N_2_ isotherms. Previously, the samples were degassed at 140 °C for 4 h under vacuum.

The silver concentration on g-C_3_N_4_ was confirmed by inductively coupled plasma optical emission spectrometry (ICP-OES Varian Vista-MPX CCD simultaneous) and compared to the standard calibration curve prepared with Ag. The analysis was repeated three times, and blank tests were conducted using the same procedure. Photoluminescence spectra were acquired at room temperature on a photoluminescence spectrometer PerkinElmer LS50B (PerkinElmer, Shanghai, China). The optical properties of the photocatalyst were studied by diffuse reflectance UV–Visible spectroscopy (DRS) using a Cary 100 spectrophotometer (Agilent Technologies, Mexico City, Mexico) in the wavelength range of 200–800 nm at a resolution of 0.1 nm. The bandgap energy values of the samples were estimated using the Kubelka–Munk (Equation (1)), as follows [24,30]:

(1) $${\left(F(R)h\vartheta \right)}^{n}=k\left(h\vartheta -E_{g}\right),F(R)={\left(1-R\right)}^{2}/2R$$

where R corresponds to reflectance, h is the constant of Plank, ϑ is the light frequency, k is a constant, $E_{g}$ is the bandgap energy, and n is a coefficient whose value is 2 for a direct bandgap or 0.5 for an indirect bandgap. The $E_{g}$ value was determined by extrapolating the linear portion of the ${\left(F(R)h\vartheta \right)}^{n}$ versus hϑ.

### 2.3. Photocatalytic Test

The photocatalytic activity of the synthesized samples was evaluated by testing their ability to degrade methylene blue (MB, Sigma-Aldrich). An MB dye solution (200 mL) with a concentration of 20 mg·L^−1^ was added to an annular-type vertical reactor to conduct the experiment. The photocatalyst loading was 1 g·L^−1^ and completely dispersed within the reactor under continuous stirring and air supply conditions. A visible lamp (Tecnolite F8T5D, 8 W nominal power) was used as the radiation source and immersed in the reactor to form the annular space reaction. The temperature of the MB solution was kept constant at 25 °C using a heating circulating bath. All experiments were subjected to dark adsorption experiments for 30 min to achieve a sample adsorption–desorption equilibrium. The identification of MB (663 nm) during photo-degradation was carried out by UV–Vis spectroscopy (Cary 100 UV–Vis). Aliquots were removed from the reaction system, centrifuged, and filtered using a membrane filter of 0.45 μm pore size prior to analysis.

### 2.4. Scavenger Testing

Trapping experiments were conducted using active species to understand the degradation reaction pathway. Ammonium oxalate (AO, 10 mM), isopropanol (IPA, 20 mM), and p-benzoquinone (BZQ, 1 mM) were utilized as scavenger agents to capture photogenerated holes, hydroxyl radicals (^•^OH), and superoxide anion radicals (^•^O_2_^−^), respectively [19,20]. Each scavenger was added to the dye solution before the irradiation, followed by the conventional dye degradation testing.

## 3. Results and Discussion

### 3.1. Characterization Results

**Figure 1:**
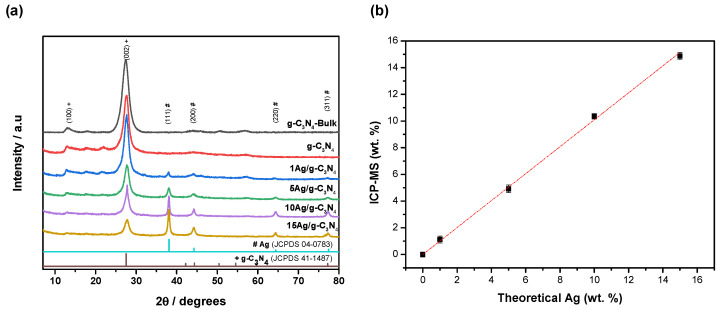
(**a**) X-ray diffraction patterns of g-C_3_N_4_-Bulk, g-C_3_N_4_, and xAg/g-C_3_N_4_ nanomaterials (+ and # correspond to g-C_3_N_4_, and Ag indexed peaks). (**b**) Chemical composition of the photocatalysts obtained by ICP-MS compared with theoretical content.

The X-ray diffraction pattern of bare g-C_3_N_4_ (bulk and thermoexfoliated) and the as-prepared xAg/g-C_3_N_4_ photocatalysts with different Ag contents are shown in Figure 1a. The diffraction pattern of the g-C_3_N_4_-Bulk material exhibits the characteristic profile of the layered g-C_3_N_4_ structure (JCPDS card 41-1487), with the interlayer-stacking (002) reflection located at a 2θ value of 26° being the most prominent. Additionally, the (100) reflection, related to the intralayer structural packing motif of tri-s-triazine units, is detected in all samples at around 13.1°. The diffractograms obtained from the thermoexfoliated materials (g-C_3_N_4_ and xAg/g-C_3_N_4_ materials) also show the (100) and (002) planes. However, all samples exhibit a slight shifting of the (002) plane, attributed to the layer exfoliation of the nanosheets caused by the second thermal treatment [1,32]. Apart from the g-C_3_N_4_ diffraction peaks, XRD patterns of the Ag/g-C_3_N_4_ materials show other diffraction peaks linked to (111), (200), and (220) planes of the FCC structure of metallic Ag (JCPDS card 04-0783). The intensity of these characteristic Ag peaks gradually increases with the metal loading from 1 to 15 wt.%, indicating that g-C_3_N_4_ and Ag coexist in the photocatalyst.

Inductively coupled plasma mass spectrometry (ICP-MS) is a technique that provides a convenient way to determine sub-nanogram-per-liter concentration levels of low-abundance elements and metal speciation [33,34]. Figure 1b shows the chemical composition analysis of the Ag/g-C_3_N_4_ photocatalysts performed via ICP-MS measurements. The results demonstrate the successful addition of different quantities of Ag over the g-C_3_N_4_ nanomaterials. Linear correlations are obtained between theoretical (synthesis conditions) and real loadings of our materials. These results agree with RDX analysis since the peak intensity of the patterns increased as the Ag content increased from bare g-C_3_N_4_ to 1%, 5%, 10%, and 15%.

**Figure 2:**
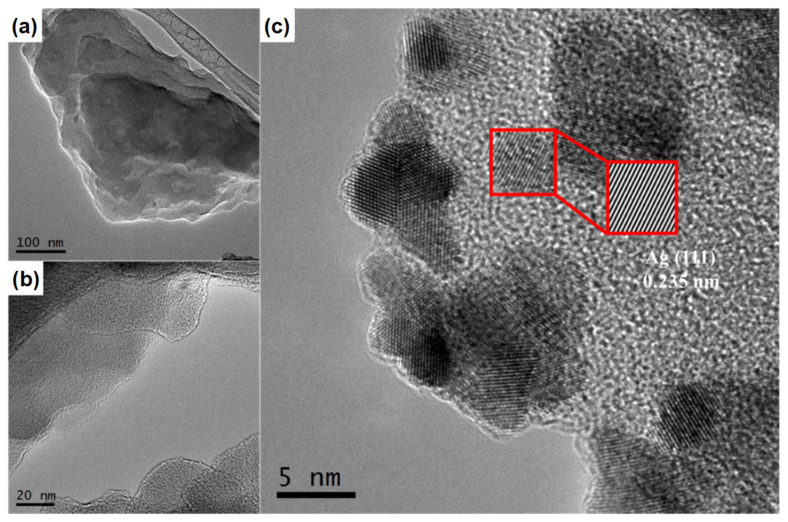
HRTEM images of (**a**) bulk g-C_3_N_4_, (**b**) thermoexfoliated g-C_3_N_4_, and (**c**) 5Ag/g-C_3_N_4_ photocatalysts.

Figure 2 presents low- and high-magnified micrographs of the g-C_3_N_4_-Bulk, thermoexfoliated g-C_3_N_4_, and most active xAg/g-C_3_N_4_ material. As seen, g-C_3_N_4_-Bulk shows the typical flat, aggregated, and non-porous flakes of the bulk-graphitic carbon nitride (Figure 2a) [1,28]. In addition, Figure 2b shows thinner layers with a rough surface and layered detachments, confirming the effect of the thermal exfoliation on the prepared g-C_3_N_4_ material. Figure 2c exhibits that the sheet structures remain intact after the deposition of Ag nanoparticles, which are homogeneously distributed. The diffraction patterns prove that the Ag nanoparticles exposed the most stable (111) plane to the surface, which agrees with the results obtained by XRD. The Ag metallic particles have a spherical shape with an average size of 6.3 nm, which could be beneficial for photocatalysis. Due to this, Ag nanoparticles can act as an electron trap. Moreover, it is appreciated that some Ag forms agglomerate in larger-sized nanoparticles. These results confirm the successful obtention of the nanocomposite material.

**Figure 3:**
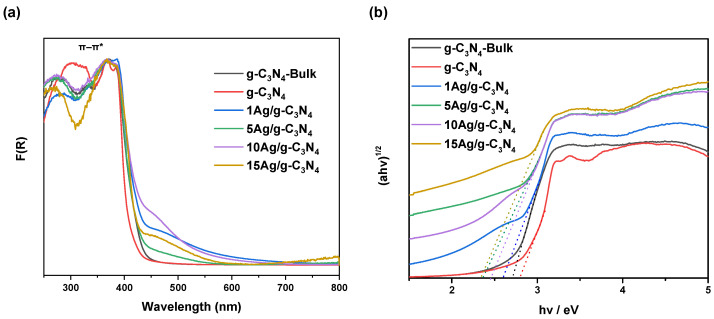
(**a**) UV–Vis diffuse absorption spectra and (**b**) (ahv)^1/2^ plots of the photocatalytic xAg/g-C_3_N_4_ and reference nanomaterials (dotted lines corresponding to applied Tauc approximation).

The UV–Vis diffuse absorption spectra of the bare g-C_3_N_4_ (bulk and thermoexfoliated) and xAg/g-C_3_N_4_ materials are shown in Figure 3. All samples show absorption peaks around 300–400 nm, assigned to π–π* transitions of the conjugated ring systems, including heterocyclic aromatics (Figure 3a). Upon comparing the xAg/g-C_3_N_4_ sample spectra with those of the reference materials, it is observed that the g-C_3_N_4_-Bulk and g-C_3_N_4_ show strong absorption in UV with an absorption onset at around 435 nm. In contrast, the xAg/g-C_3_N_4_ materials showed strong and extended absorption in the visible-light region and a red shifting of the absorption edge, which increases as the amount of silver over the thermoexfoliated g-C_3_N_4_ rises. In addition, the formation of an absorption band around 480 nm is appreciated, attributed to the plasmonic properties of the Ag nanoparticle agglomerates of a larger size (>10 nm) [29]. This is in accordance with results obtained by TEM, where some Ag nanoparticles are agglomerates that form secondary nanoparticles of a larger size.

Figure 3b shows that the influence of the addition of silver on g-C_3_N_4_ can be reflected in the bandgap values obtained by the Tauc method [30]. The bandgap values for g-C_3_N_4_-Bulk and g-C_3_N_4_ were estimated to be 2.78 and 2.74 eV, respectively. In the case of xAg/g-C_3_N_4_ nanocomposites, bandgap values decreased from 2.63 eV (1Ag/g-C_3_N_4_) to 2.36 eV (15Ag/g-C_3_N_4_) as the content of Ag increased, as summarized in Table 1. These results indicate that adding Ag to g-C_3_N_4_ enhances the materials’ ability to absorb photons, which is beneficial for generating reactive oxygen species (ROS) and, therefore, the photocatalytic process.

**Figure 4:**
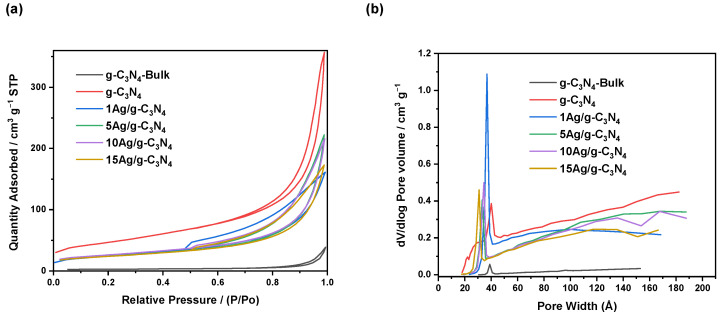
(**a**) N_2_ adsorption and desorption isotherms and (**b**) pore size distribution of the photocatalytic xAg/g-C_3_N_4_ and reference nanomaterials.

Figure 4 and Table 1 show the textural properties of the g-C_3_N_4_-Bulk, g-C_3_N_4_, and xAg/g-C_3_N_4_ photocatalytic materials. Figure 4a displays N_2_ adsorption–desorption isotherms and pore size distribution (Figure 4b) of the nanomaterials. According to the IUPAC classification, the isotherms of the samples (Figure 4a) correspond to isotherm type IV with apparent H3 hysteresis loops, indicating the existence of a mesoporous structure [35]. Table 1 summarizes the textural values of the photocatalysts, where the results reveal that the thermoexfoliation treatment applied to the g-C_3_N_4_-Bulk material increases the specific surface area from 9.2 to 143.8 m^2^ g^−1^ (g-C_3_N_4_). This result validates the effectiveness of the technique in obtaining more g-C_3_N_4_ nanosheets with a high specific surface area that can lead to better light absorption and accessibility to reactant molecules during the photocatalytic reaction. Regarding the as-prepared xAg/g-C_3_N_4_ materials, the incorporation of Ag nanoparticles into g-C_3_N_4_ generated a decrease of 40% (average) compared to g-C_3_N_4_. However, a trend of increasing or decreasing the specific surface area is not appreciated as silver concentration increases. Although considerable, the decrease in specific surface area is not significant, especially when considering the improvement in bandgap width obtained for all silver-containing materials. In the case of pore volume results, Figure 4b presents a monomodal distribution where the pore size decreased as the Ag content increased over the g-C_3_N_4_ surface, indicating that the deposition of Ag can block the pores of g-C_3_N_4_. Here, the pore volume value obtained for thermoexfoliated g-C_3_N_4_ (0.257 cm^3^ g^−1^) is higher than that obtained for the g-C_3_N_4_-Bulk (0.013 cm^3^ g^−1^). The values obtained for the samples that contain Ag nanoparticles are in the same order of magnitude (average of 0.182 cm^3^ g^−1^), without an increasing or decreasing trend of the silver concentration. Additionally, the textural properties of the g-C_3_N_4_ and Ag/g-C_3_N_4_ materials are more promising than those of the typical titania used as a reference in photocatalytic studies [31].

### 3.2. Photocatalytic Activity Results

**Figure 5:**
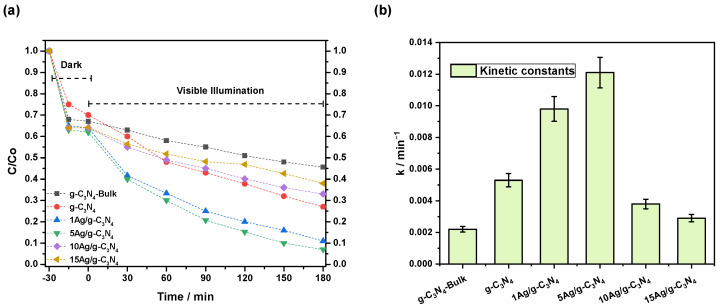
(**a**) Photocatalytic degradation of methylene blue and (**b**) kinetic constant, obtained with the xAg/g-C_3_N_4_ and reference nanomaterials.

The photocatalytic experiments of g-C_3_N_4_-Bulk, g-C_3_N_4_, and Ag/g-C_3_N_4_ were evaluated via methylene blue degradation under visible-light irradiation. Figure 5a displays the concentration ratio C/Co as a function of reaction time, including a stabilization period under dark conditions. It is essential to highlight that in the absence of the photocatalyst, the degradation of MB is negligible after 180 min. Furthermore, it is observed that the photocatalysts present adsorption of the dye pollutant by ca. 25% to 30% under dark conditions, indicating that the adsorption ability of Ag/g-C_3_N_4_ towards MB molecules slightly enhances with Ag loading. The photodegradation results (visible illumination zone) show that the thermoexfoliation process allows for increasing the methylene blue remotion in the order of 20% more than the g-C_3_N_4_-Bulk material. Likewise, it can be seen that silver deposited on g-C_3_N_4_ thermoexfoliated modifies the photocatalytic behavior of the exfoliated graphitic carbon nitride nanosheets. Moreover, 1Ag/g-C_3_N_4_ and 5Ag/g-C_3_N_4_ photocatalysts managed to eliminate 89 and 94% of the methylene blue in 180 min. However, the incorporation of 10 and 15% of Ag nanoparticles decreases the photoactivity, obtaining less methylene blue remotion than the g-C_3_N_4_ material. The first-order kinetic constant was determined to evaluate the real photocatalytic activity of each nanomaterial, and the results are shown in Figure 5b. The value obtained for the thermoexfoliated graphitic carbon nitride (g-C_3_N_4_) is 2.6 times higher than that obtained with the g-C_3_N_4_-Bulk photocatalyst (0.002 min^−1^). In addition, as can be seen, the incorporation of 1 (0.010 min^−1^) and 5 (0.012 min^−1^) wt.% of silver nanoparticles improves the photocatalytic activity, obtaining the best performance with the 5Ag/g-C_3_N_4_ photocatalysts. Therefore, the photocatalytic results indicate that an optimum amount of silver enhances the adsorption of the colorant under dark conditions and its degradation under visible-light illumination.

**Figure 6:**
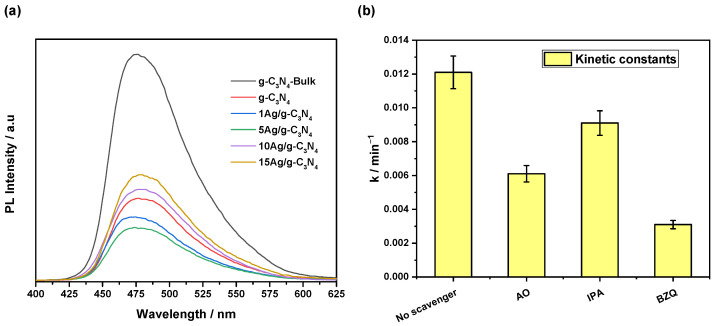
(**a**) Photoluminescence (PL) spectra of the photocatalyst samples. (**b**) Effect of different scavengers on the reaction rate of the 5Ag/g-C_3_N_4_ sample.

To elucidate the photoactivity improvement effect of the xAg/g-C_3_N_4_ photocatalysts concerning the g-C_3_N_4_-Bulk material, as well as the differences in activity between the materials of the series, a photoluminescence (PL) spectroscopy study was carried out to correlate the improvement with the inhibition effect of charge carrier recombination (e^−^/h^+^), and the results are shown in Figure 6a. It is observed that each of the spectra presents a similar shape characteristic of g-C_3_N_4_, with slight differences in the maximum position located between 440 nm and 550 nm, corresponding to electronic transitions between bands and localized states [15,36]. However, there are significant differences in the intensity of the spectra. As is known, a higher intensity of PL signals corresponds to a higher recombination rate of charge carriers [37,38]. In this regard, low PL intensity is preferred for using the material as a photocatalyst [39,40]. Considering the latter, the results in terms of intensity show a direct correlation with the photoactivity results (see Figure 5b), i.e., the photocatalyst with the best performance (5Ag/g-C_3_N_4_) displays a lower signal intensity, followed by 1Ag/g-C_3_N_4_ < g-C_3_N_4_ < 10Ag/g-C_3_N_4_ < 15Ag/g-C_3_N_4_ < g-C_3_N_4_-Bulk. This tendency indicates that the photocatalytic behavior of the materials is dominated by the decrease in the recombination of electron-hole pairs attributed to the thermal-exfoliation process and the addition of the optimal amounts of Ag.

In a typical degradation-by-photocatalysis process, the photogenerated holes, electrons, and superoxide (^•^O_2_^−^) and hydroxyl (^•^OH) radicals play a crucial function in the degradation of organic contaminants. Figure 6b displays the effect of scavengers on methylene blue degradation using BZQ, AO, and IPA as scavengers of ^•^O_2_^−^, holes, and ^•^OH radicals, respectively, under visible-light radiation. These reactive species are used to elucidate the role of the reactive species on MB degradation. As can be seen, the 5Ag/g-C_3_N_4_ sample presents 94% MB degradation without adding any scavenger. However, the addition of BZQ inhibited the performance significantly. This result denoted that ^•^O_2_^−^ is the main reactive species that carries out the photocatalytic activity [41]. Also, a decrease in photoactivity for 5Ag/g-C_3_N_4_ was observed in the presence of AO, which indicated that the photogenerated holes also contributed to the photocatalytic process. Herein, the photogenerated electrons could react with molecular oxygen absorbed on the surface of 5Ag/g-C_3_N_4_ to produce ^•^O_2_^−^ radicals. On the other hand, it is known that the standard redox potential of the g-C_3_N_4_ valence band is more negative than ^•^OH/OH^−^; consequently, holes in the valence band of g−C_3_N_4_ could not react with molecules to form ^•^OH radicals [42,43]. This effect agrees with our results, where the degradation performance presents a non-significant change with IPA addition, indicating the poor contribution of ^•^OH radicals.

**Figure 7:**
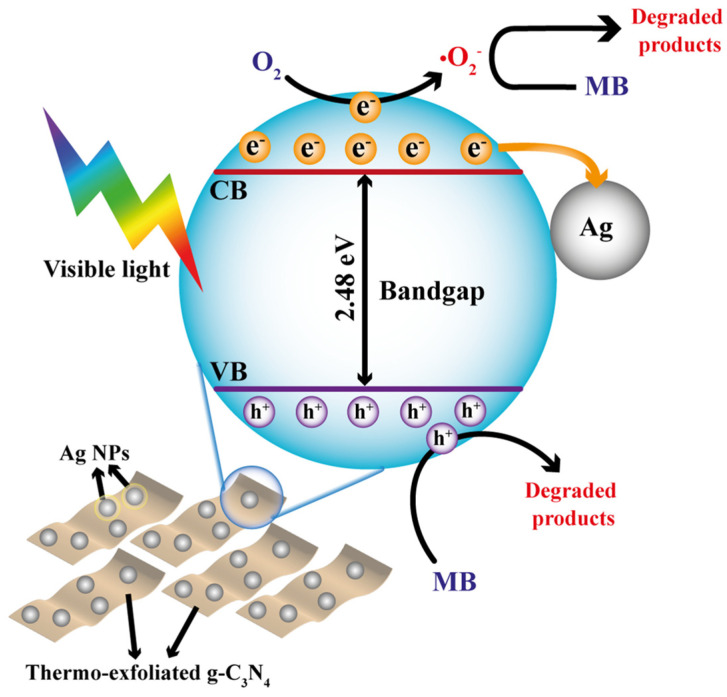
Proposed schema for the enhanced photoactivity of the Ag/g-C_3_N_4_ photocatalyst under visible-light radiation.

Based on the above analysis, a schematic diagram of a possible photocatalytic mechanism for a 5Ag/g-C_3_N_4_ photocatalyst is displayed in Figure 7. It is known that the photocatalytic activity of a material is influenced by its textural, structural, optical, and electronic properties. In our work, the enhanced performance of photocatalyst degradation was mainly attributed to the synergistic effect of thermoexfoliated g-C_3_N_4_ with a superior surface area and the presence of Ag nanoparticles over g-C_3_N_4_ sheets. The obtained exfoliated g-C_3_N_4_ nanosheets present superior textural characteristics (surface area of 143.8 m^2^ g^−1^), which could provide more active sites for redox reactions and better photoactivity. Moreover, the migration distance of charges from the bulk to the surface was significantly decreased in g-C_3_N_4_ after exfoliation, resulting in a decreased recombination process during the migration of the photogenerated charges to the surface. Moreover, the photocatalytic activity of the material containing 5 wt.% of Ag exhibits the primary role of silver nanoparticles, acting as electron traps to decrease charge recombination. However, new charge-recombination centers are generated at higher Ag contents than the optimal amount, resulting in a decrease in photoactivity [44]. This result is supported by PL analysis, where exfoliated g-C_3_N_4_ samples showed a significant reduction in the intensity band of PL compared to bulk g-C_3_N_4_. This last observation is even more evident with the incorporation of Ag. This effect can be explained by the good dispersion of the Ag nanoparticles in g-C_3_N_4_, which assisted in improving the number of photocatalytic sites. However, introducing large concentrations of metal to a photocatalyst can cause an agglomeration of particles and a loss of active surface.

Based on the above discussion, this work denotes the effectiveness of the thermoexfoliation methodology used to obtain g-C_3_N_4_ with a high surface area. In this regard, Table 2 summarizes the degradation performance of Ag/g-C_3_N_4_ produced by two-step thermoexfoliation. As can be seen, the Ag/g-C_3_N_4_ composite obtained in this work presents a higher surface-area value (90.0 m^2^ g^−1^). In the best case, the photocatalytic performance is similar or superior to those obtained using the methodologies using UV or visible lamps as radiation sources. This fact denotes the suitability of obtaining Ag/g-C_3_N_4_ composites via thermoexfoliation as a helpful method for wastewater remediation.

## 4. Conclusions

In the present work, high-surface-area Ag/g-C_3_N_4_ photocatalysts with different loads of Ag (1, 5, 10, and 15 wt.%) were prepared by a thermal-exfoliation process, and their photocatalytic activity was evaluated through MB degradation. The thermoexfoliation process was very effective in increasing the number of nanosheets as well as the surface area of g-C_3_N_4_-Bulk. The results showed a significant increment in the surface area of thermally exfoliated g-C_3_N_4_ (143.8 m^2^ g^−1^) compared to g-C_3_N_4_-Bulk (9.2 m^2^ g^−1^). Furthermore, the incorporation of Ag affects the photocatalytic activity. The best performance was obtained with the 5Ag/g-C_3_N_4_ nanomaterial, which removed the 94% MB dye in 180 min. The radical scavenger test revealed that ^•^O_2_^−^ and holes were the main species responsible for the degradation process. The enhanced performance was attributed to the decrease in charge-recombination processes resulting from the combined effect of the superior textural properties of the exfoliated g-C_3_N_4_ as well as Ag loading. These results indicate that these g-C_3_N_4_-based nanomaterials could be suitable for water remediation under sunlight irradiation.

## Figures and Tables

**Table 1:** Textural properties and Eg values of g-C_3_N_4_-Bulk, g-C_3_N_4_, and Ag/g-C_3_N_4_ nanomaterials.

Catalyst	BET Surface Area (m^2^·g^−1^)	Pore Volume (cm^3^·g^−1^)	Average Pore Size (nm)	Bandgap (eV)
g-C_3_N_4_-Bulk	9.2	0.013	6.9	2.74
g-C_3_N_4_	143.8	0.257	5.2	2.78
1Ag/g-C_3_N_4_	85.9	0.189	5.6	2.63
5Ag/g-C_3_N_4_	88.9	0.186	6.3	2.48
10Ag/g-C_3_N_4_	89.9	0.180	5.9	2.43
15Ag/g-C_3_N_4_	87.8	0.179	6.1	2.36

**Table 2:** Photocatalytic activity of Ag-modified g-C_3_N_4_ prepared under different methodologies.

Sample	Methodology	Surface Area [m^2^ g^−1^]	Application	Light Source	Ref.
Ag-Modified g-C_3_N_4_	One-step calcination at 550 °C for 8 h)	31.5	MO degradation (98.7% in 2 h)	Visible (300 W Xe lamp with a 420 nm filter)	[45]
Ag@g-C_3_N_4_	Single-step process at 550 °C for 4 h	38.1	MB (100%, 210 min) and RhB degradation (~89%, 250 min)	Visible (3M, 400 W, λ > 500 nm)	[46]
Ag/g-C_3_N_4_ composite	Thermal treatment at 550 °C for 4 h	7.3	MG degradation (80%, 100 min)	UV (40 W, Philips)	[47]
Ag/g-C_3_N_4_	Thermal polymerization at 250 °C for 1 h, 350 °C for 2 h, and 550 °C for 2 h	66.0	MO degradation (92%, 120 min)	Visible (Xe lamp, 300 W with UV and IR filters)	[48]
Ag/g-C_3_N_4_	Thermal exfoliation at 500° for 2 h, and 500 °C for 2 h	90.0	MB degradation (94%, 180 min)	Visible (8 W, Tecnolite)	Present work

## Data Availability

Data are contained within the article.
